# Selection of *Neospora caninum *antigens stimulating bovine CD4^+ve ^T cell responses through immuno-potency screening and proteomic approaches

**DOI:** 10.1186/1297-9716-42-91

**Published:** 2011-08-03

**Authors:** Mara S Rocchi, Paul M Bartley, Neil F Inglis, Esther Collantes-Fernandez, Gary Entrican, Frank Katzer, Elisabeth A Innes

**Affiliations:** 1 Moredun Research Institute, Pentlands Science Park, Bush Loan, Midlothian, EH26 0PZ, UK; 2 SALUVET, Animal Health Department, Faculty of Veterinary Sciences, Complutense University of Madrid, Avda Puerta de Hierro s/n, 28040 Madrid, Spain

## Abstract

*Neospora caninum *is recognised worldwide as a major cause of bovine infectious abortion. There is a real need to develop effective strategies to control infection during pregnancy which may lead to either abortion or congenital transmission. Due to the intracellular nature of the parasite, cell-mediated immune (CMI) responses involving CD4^+ve^, CD8^+ve^, γ/δ TCR^+ve ^T cells and NK cells, as well as production of IFN-γ, are thought to be important for protective immunity. In this study we applied a combination of proteomic and immunological approaches to identify antigens of *N. caninum *that are recognized by CD4^+ve ^T cell lines derived from infected cattle. Initially, *N. caninum *tachyzoite Water Soluble Antigens (NcWSA) were fractionated by size-exclusion HPLC and then screened for immune-potency using CD4^+ve ^T cell lines. LC-ESI-MS/MS (liquid chromatography electrospray ionisation tandem mass spectrometry) was employed to catalogue and identify the proteins comprising three immunologically selected fractions and led to the identification of six *N. caninum *target proteins as well as sixteen functional orthologues of *Toxoplasma gondii*. This approach allows the screening of biologically reactive antigenic fractions by the immune cells responsible for protection (such as bovine CD4^+ve ^cells) and the subsequent identification of the stimulating components using tandem mass spectrometry.

## Introduction

*Neospora caninum *is a protozoan parasite, closely related to *Toxoplasma gondii*, which has emerged as a major cause of reproductive failure in cattle worldwide [1,2]. The parasite is now recognised as the most commonly diagnosed cause of abortion in areas with an intensive dairy industry [3]. Infection during pregnancy may result in abortion, depending on the stage of gestation when parasitaemia occurs, or may lead to the birth of a congenitally infected calf [4]. Treatment options are limited, with few chemotherapeutics available which may be problematic to use in meat or milk-producing livestock. Applying management and biosecurity measures such as those detailed in a management scheme recently launched by Defra in the UK (Herdsure) [5], may help to reduce infection levels in the herd; culling of seropositive animals has also been suggested as a method of control [6]. All these approaches can constitute a substantial cost for the farming industry.

There is accumulating evidence that cattle previously exposed to the parasite are less likely to abort than those undergoing a primary infection [7] suggesting the development of some form of protective immunity and the feasibility of a vaccination approach. To date only one commercial vaccine [8], based on an inactivated tachyzoite preparation adjuvated with Havlogen [9], has been registered in some countries. This vaccine demonstrated variable reduction in the number of abortions under field challenge condition in Costa Rica [10] and New Zealand [11]. However, it did not prevent foetal infection [12] and did not allow discrimination between vaccinated and naturally infected animals. Studies that have focussed on the evaluation of *N. caninum *tachyzoite proteins as vaccine candidates in mouse models have given ambiguous results, ranging from 70-90% protection using live attenuated tachyzoites [13] to very little or no protection with the SRS2 antigen and ISCOMs [14]. It appears that immunisation with live attenuated organisms is more effective than killed organisms, presumably as a reflection of more efficient antigen processing and presentation to T cells.

Immunological screening requires knowledge of the immune mechanisms responsible for protection, the so-called correlates of protection [15]. Cattle infected with *N. caninum *produce parasite-specific antibodies although their contribution to protective immunity is not clear [7,16]. There is mounting evidence that, as for other intracellular protozoan parasites, the most important correlate of protection for *N. caninum *is the establishment of a cell mediated immune response [7,17]. *In vitro *studies have shown that treatment of cultured cells with recombinant interferon gamma (IFN-γ), a cytokine produced by activated T-lymphocytes, significantly inhibits the intracellular multiplication of *N. caninum *[18]. A number of studies have also demonstrated that activated T-lymphocytes can recognise and respond to parasite-infected cells by producing inhibitory cytokines [19,20]. Staska et al. have shown a T-helper type 1 response in infected cattle involving CD4^+ve ^cytotoxic T cells and IFN-γ production [21], whereas Boysen et al. showed that cytotoxic NK cells also play a role in the control of the disease through both cytotoxic and an IFN-γ mediated mechanisms [22]. Therefore, the development of vaccines directed against *N. caninum *should focus on selecting antigens that are capable of eliciting mainly a cell mediated immune response involving CD4^+ve ^T cells and IFN-γ, in addition to a serological response.

The aim of this work was to identify *N. caninum *tachyzoite antigens that are recognised by the cell-mediated immune (CMI) response of experimentally infected animals. *Neospora caninum *water soluble antigens were initially separated by size exclusion HPLC and tested for their ability to induce proliferative responses in a NcWSA-specific bovine CD4^+ve ^test system. A number of fractions which consistently induced significant proliferative responses were further investigated by tandem mass spectrometry allowing the identification of the proteins present. This type of approach demonstrated that is possible to use biologically relevant screening tools to select T-cell reactive fractions, thus facilitating the downstream analysis of relevant candidate vaccine antigens for *Neospora caninum*.

## Materials and methods

### Experimental design

*Neospora caninum *water-soluble antigen (NcWSA) was subdivided into smaller, less complex protein pools by size exclusion HPLC. Three identical aliquots of the same preparation of NcWSA were run in three consecutive size exclusion separations and tested *in vitro *for immune recognition by short-term NcWSA specific CD4^+ve ^cell lines derived from cattle experimentally infected with *N. caninum*. Immuno-reactive fractions were subjected to SDS-PAGE and LC-ESI-MS/MS prior to downstream database mining and bioinformatic analysis to identify their respective protein compositions.

### Parasites, inocula and immunisation schedules

*Neospora caninum *tachyzoites (NC1 isolate) [23] were maintained in Vero cells as previously described [18] and used to prepare infectious inocula as detailed below. Experimental live tachyzoite challenge confers protection against abortion [24] and has been employed in the past by us and other groups to characterise protective immune responses [25,26]. Calves (see below) were infected subcutaneously over the left pre-femoral lymph node with the live inoculum containing 1 × 10^8 ^tachyzoites per calf. A control inoculum containing an equivalent number of Vero cells as present in the parasite inocula was administered to control calves. All experimental animals employed in this study were reared, housed and handled in accordance to the UK Animals (Scientific Procedures) Act 1986; the experimental design was approved by the Moredun Ethical Review Committee. Five male calves, dehorned and castrated, aged two months and serologically negative for *N. caninum *antibodies by IFAT [27] and a commercial ELISA (NC Herdcheck, IDEXX Laboratories, Chalfont St Peter, UK) were randomly assigned to two groups. Three animals received the infectious inoculum whereas the two remaining animals received the control. Rectal temperatures were monitored from day 2 to day 14 post-infection (pi) and blood samples were collected up to one month post-infection for serological analysis. Seroconversion was confirmed by ELISA. Twelve months after the first inoculation the animals were boosted with a further similar dose of either live *N. caninum *tachyzoites or control Vero cell inoculum.

### Lymphocyte Transformation Tests (LTT)

Initiation of a CMI response was confirmed by LTT (Lymphocyte Transformation Test) on Peripheral Blood Mononuclear Cells (PBMCs) isolated according to previously published protocols [28]. Briefly, PBMCs were resuspended in cell culture medium (CCM) (comprising IMDM [Gibco, Invitrogen, Paisley, UK], 10% heat inactivated foetal bovine serum (FBS), 2 mM L-glutamine, 100 U/mL penicillin, 100 μg/mL streptomycin and 5 μg/mL Amphotericin B [all from Sigma, Gillingham, UK]). PBMCs were cultured in 96-well plates (Nunc, Roskilde, Denmark) at concentration of 2 × 10^5^/well and stimulated with 200 ng/well of antigen (NcWSA-see below) for 5 days in a humidified 5% CO_2 _atmosphere at 37°C. Controls included cell culture media, Concanavalin A (ConA) (1 μg/well, Sigma, UK) and Vero cell lysate (200 ng/well). Cultures were pulsed with 1 μCi of [^3^H]-thymidine (GE HealthCare, Bucks, UK) for the final 18 h of incubation. Cells were harvested onto glass fibre filters (Wallac, Turku, Finland) and [^3^H]-thymidine incorporation quantified using an automated scintillation counter (Perkin Elmer, Cambridge, UK) and expressed as counts per minute (CPM), with each test performed in quadruplicate. Stimulation indices (SI) were calculated by dividing the median value of the test by the median value of the media control.

### Preparation of *N. caninum *Water-Soluble Antigen (NcWSA)

NcWSA was produced as follow: 1-2 × 10^9 ^tachyzoites, prepared accordingly to previously published methods [29] were washed three times in PBS (650 × *g *for 5 min) then stored at -20°C prior to antigen preparation. After thawing, tachyzoites were suspended in distilled water and disrupted by three cycles of freezing and thawing in liquid nitrogen followed by homogenisation using a Precellys tissue homogenizer (Precellys, Bertin Technologies, Tarnos, France). The homogenised suspension was centrifuged at 10 000 × *g *for 30 min at +4°C to recover the supernatant containing the *N. caninum *WSA. Protein concentration was assessed using the BCA reagent (Pierce Chemicals, Rockford IL, USA). NcWSA was then aliquotted and stored at +4°C prior to chromatographic fractionation, which was performed within 24 h of antigen preparation.

### Size exclusion HPLC of NcWSA

Size exclusion chromatography was performed using a Beckman System Gold HPLC apparatus (Beckman Coulter, High Wycombe, UK) in combination with a Superose 12 gel filtration column (GE Healthcare) pre-equilibrated with PBS pH 6.8. Individual 200 mL injections of 0.45 mm-filtered NcWSA (935 mg total protein) were applied to the column and the proteins resolved isocratically in PBS pH 6.8 at a flow rate of 0.5 mL/min over a period of 60 min. Proteins eluting from the column were monitored by UV (280 nm) and chromatographic data was recorded and analysed using 32 Karat Gold ™ chromatograpy analysis software (Beckman Coulter). Fractions of 1.0 mL were collected and stored at +4°C in sealed low-protein-binding tubes (Eppendorf, Cambridge, UK) until required. Reproducibility of the fractionation was confirmed by overlaying the chromatograms of three consecutive separations as shown in results.

Protein quantification of individual antigen fractions was performed using the NanoOrange^® ^protein quantification kit (Molecular Probes, Invitrogen, Paisley, UK) in accordance with the manufacturer's instruction and an automated fluorescence reader (CytoFluor, PerSeptive Biosystems, Framingham, MA, USA). Protein concentrations were then adjusted between fractions to ensure equal concentration of each fraction used in the T-cell lines proliferation assays, and fractions were stored at -20°C until required for the T cell assay.

### Generation and characterization of bovine CD4^+ve ^T cell lines and antigen screening assay

Short-term antigen specific T cell lines were prepared from the infected animals as follows: for the first round of stimulation each well of a 96-well round bottom tissue culture plate (Nunc, Denmark) was seeded with 2 × 10^5 ^freshly isolated PBMCs resuspended in CCM and stimulated with 200 ng NcWSA. Cells were cultured at 37°C in a humidified 5% CO_2 _atmosphere for seven days then harvested, washed (300 × *g *for 10 min), resuspended in CCM supplemented with 10 U/mL of recombinant human IL-2 (rhu IL-2, Proleukin, Novartis, East Hanover NJ, USA) and seeded into fresh 96-well round bottom plates. Six days later an aliquot of cells was removed for phenotypical analysis and the remaining cells were cultured for a further 24 h prior to use in the antigen screening assay. The phenotypic composition of the short-term T cell lines was analysed using a panel of monoclonal antibodies (MoAbs) recognizing specific bovine leukocyte populations according to previously published methods [30]. Antibody binding was revealed with Alexa 488-conjugated anti-mouse IgG (Invitrogen, UK) (0.5 μg/mL final) and data acquired using a CyAn flow cytometer (CyAn, Dako-BeckmanCoulter, USA) equipped with a 488 nm argon-ion laser and analyzed using Summit software (Dako, Fort Collins, CO, USA). A minimum of 10 000 cells were acquired for each sample.

These short term lines prepared from the three infected animals were used to test each fraction generated from the three different HPLC runs (*n *= 9). Test wells for the T cell lines screening assays were set up in triplicate in 96 well round bottom tissue culture plates. Each well contained 5 × 10^4 ^T cells, 5 × 10^5 ^autologous antigen presenting cells (APC) (3000 rad γ-irradiated PBMCs; at a 1:10 ratio) and the different HPLC-separated fractions (1-25) at a final concentration of 10 ng/well. Negative controls comprised T cells or APCs with medium only as well as APCs plus T cells with only CCM or with Vero lysate. Positive controls included T cells, APCs or APCs plus T cells cultured with 500 ng/well ConA or unfractionated NcWSA with a final concentration comprised between 200 and of 10 ng/well. Cells were cultured at 37°C in a humidified 5% CO_2 _atmosphere and proliferation was quantified by ^3^H-thymidine incorporation as described previously for the proliferation assay.

### Shotgun proteomic analysis of selected reactive fractions

All protemics-based analysis were performed by the Moredun Proteomic Facility, (Moredun Research Institute, Penicuik, UK). A pool of each homologous fraction selected on the basis of the CD4^+ve ^T cell reactivity was dialyzed overnight against HPLC-grade water (membrane cut-off 3 kDa), snap-frozen in liquid nitrogen then freeze-dried. Each pellet was resuspended in 25 μL of reducing SDS-PAGE sample buffer, heated at 95°C for 5 min, separated using a SDS-PAGE gel (4-12% Tris-glycine gradient, NuPage, Invitrogen, UK) and finally stained with Simply Blue Safe Stain ™ (Invitrogen, UK). The gel lanes were excised in their entirety then divided equally into slices of 2.5 mm deep to yield 25 gel slices. Each slice was then de-stained before processing using standard in-gel reduction, alkylation and trypsinolysis procedures [31]. The resulting peptides were analysed by Liquid Chromatography Electrospray Ionisation Tandem Mass Spetrometry (LC-ES-MS/MS) using a U3000 nano-flow UHPLC apparatus (Dionex, Camberley, UK) and amaZon high capacity ion trap mass spectrometer (Bruker, Coventry, UK). Parameters for tandem MS analysis were set as previously described [32]. Processed MS/MS data, in mascot generic format (mgf), was mined against a) the NCBInr database [33] using alveolata as taxonomical search and b) a cognate *Neospora caninum *genomic database (N.c. Liverpool strain) [34]. The presentation and interpretation of MS/MS data was performed in accordance with published guidelines [35]. A more detailed description of the Tandem Mass Spectrometry procedure can be found in the additional file 1.

## Results

### Clinical and immunological reactivity after challenge

Between 72 and 96 h after the initial *N. caninum *challenge, the infected animals showed pyrexia and swelling of the ipsilateral draining lymph nodes, whereas the negative control animals remained normal. In the infected animals seroconversion was demonstrated 14 days post infection (dpi) by ELISA (data not shown) and antigen specific CMI responses were detected one month after infection by LTT (SI values between 14 and 135). Control animals showed no seroconversion or antigen-specific responses (SI values between 2 and 3; data not shown). Twelve months after the initial challenge the infected animals were inoculated with a second live immunisation, using a similar dose and route as described in material and methods. Following the second immunisation, LTT reactivity was evident and serological reactivity was demonstrated using Western blot analysis (an example of Western blot reactivity is shown in an additional figure - additional file 2). The animals were employed to prepare CD4^+ve ^T cell lines starting from three weeks after the second challenge.

### HPLC fractionation of *N. caninum *water-soluble antigen

**Figure 1:**
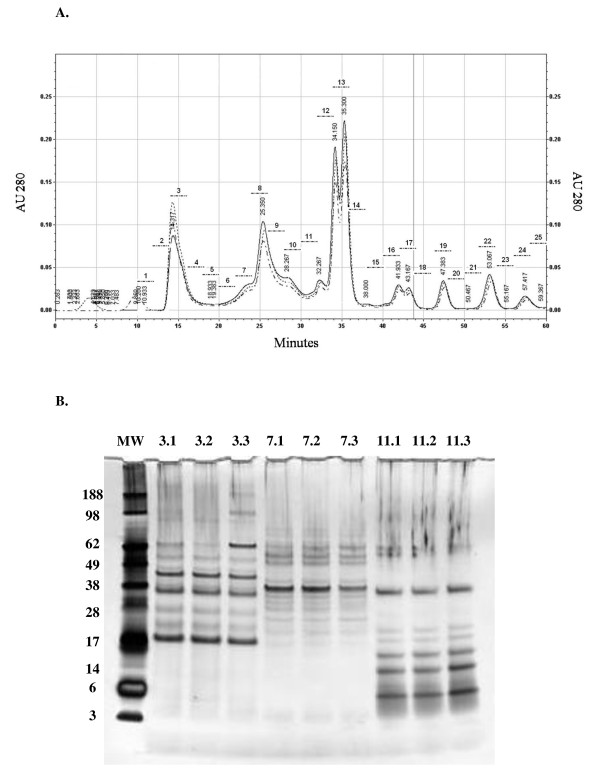
**Size exclusion fractionation of *N. caninum *Water Soluble Antigen**. (A). Three identical aliquots of NcWSA were divided into fractions comprising molecules of progressively lower mass by size exclusion HPLC. The superimposed absorbance profiles of the fractions generated in three successive runs are shown. Horizontal axis: time, vertical axis: 280 nm absorbance. Fraction collection started 10 min into the run, flow rate was 0.5 mL/min and a new fraction was collected every 2 min. The horizontal dotted lines indicate which portion of the NcWSA antigen corresponded to each collected fraction; bold numbers above the dotted lines represent the fraction number, vertical numbers represent retention time in minutes. **(B)**: Silver stained SDS PAGE gel (4-12% Bis-Tris gradient) of three representative fractions (3, 7 and 11) obtained in three separate runs (.1, .2 and .3) of size-exclusion chromatography. MW: molecular weight markers; 3.1: fraction 3 run 1; 3.2: fraction 3 run 2; 3.3: fraction 3 run 3; the numeration is equivalent for the further two fractions.

Corresponding fractions (1-25) from each of three identical size exclusion separations of NcWSA were combined to yield 25 pooled fractions. Chromatograms of each separation were superimposed to demonstrate consistency over the three replicate runs (Figure 1A). In addition, similarity between homologous fractions obtained from the three consecutive runs was confirmed by SDS-PAGE analysis on three representative fractions (Figure 1B). An example of SDS-PAGE analysis of the HPLC-separated fractions is shown in an additional figure (see additional file 3). These results demonstrate the robustness of the HPLC fractionation and show that this is independent from the size of the separated proteins.

### Antigen fraction screening using CD4+ve T cell lines

**Figure 2:**
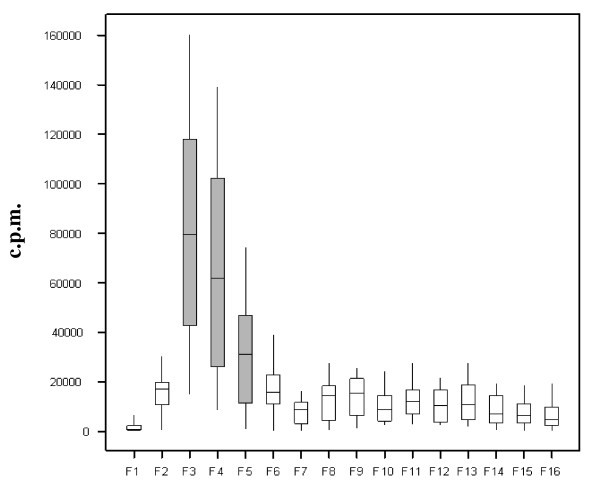
**Proliferation of CD4^+ve ^T cell lines in response to the fractionated *N. caninum *water-soluble antigen**. Average proliferative response of 9 different cell lines (three each from three animals) to the first 16 HPLC fractionated antigen preparations. Results are expressed as count per minute (cpm-vertical axis). F1-F16 represents the fraction number. Each box plot spans the interquartile range of cpm values, with the inside line indicating the median value. Whiskers extend as far as the minimum and maximum values. The fractions selected for further proteomic analysis are shadowed. Fractions 17 to 25 were not recognised or recognised at background level by the cell lines and are not represented in the figure.

Short term stimulation of immune PBMCs with NcWSA and IL-2 produced 2-week old cell lines that consistently comprised a majority of CD3^+ve ^and CD4^+ve ^T cells (CD3^+ve^: average 97%, min 94.3% and max 98.5%; CD4^+ve ^average 87%, min 77.2% and max 93.2%) with very low CD8^+ve ^and γ/δ TCR^+ve ^T cells contamination (on average less than 5% for CD8 and 8% for γ/δ TCR^+ve^). These cells were employed in the T cells assays for the antigen fraction screening test where we identified three fractions (fractions 3, 4 and 5) which consistently induced a cellular reactivity above all the other fractions tested (Figure 2). This reactivity corresponded, on the HPLC trace, to proteins collected between 14 and 20 min. from the start of the chromatographic separation. Fraction 3 showed the highest reactivity, closely followed by fraction 4, whereas fraction 5 was higher than all the remaining fractions but to a large extent lower than 3 and 4. Despite the presence of higher concentration of proteinaceous (as visualised by SDS-PAGE, additional file 3) and non-proteinaceus material (as deduced from the peaks in the HPLC trace) in other fractions, the remaining fractions were only marginally stimulatory to the T cell lines. In addition, fractions 17 to 25 consistently failed to induce reactivity above background level. On this basis, fractions 3, 4 and 5 were selected for further analysis.

### Proteomic analysis of selective reactive fractions

Proteins contained within fractions 3, 4 and 5, identified as consistently reactive with the CD4^+ve ^T cell lines, were separated by SDS-PAGE and catalogued by shotgun proteomic analysis using LC-ESI-MS/MS. For each individual fraction sample, a non-redundant list of identified proteins was prepared, and a master list was generated, which was termed as *N. caninum *reactive protein list (NCRP) which comprises a total of six unique *N. caninum *proteins (Table 1) as well as sixteen *T. gondii *homologues (Tables 2 and 3). Surface antigens, proteasome subunits, microneme and dense granule proteins as well as some putative uncharacterised proteins were identified. Three of the *N. caninum *proteins were present both in fractions 3 and 4 (SAG1, SRS2 and GRA2), two were found exclusively in fraction 3 (microneme protein Nc-MIC3 and GRA7) and only one was identified exclusively in fraction 4 (microneme protein NcMIC11). Homologues of *T. gondii *proteins identified comprised surface, ribosomal, proteasome, histone and rhoptry proteins, seven of which were identified in fraction 3, nine in fraction 4 and three in fraction 5. In some cases the same protein was indentified in contiguous fractions (Table 2) however, the majority of *T. gondii *homologues were present exclusively in a single fraction (Table 3).

**Table 1:** NCRP list of *N. caninum *proteins identified in reactive fractions

Fraction(s)	Accession	Gene name	Description	MOWSE	Peptides Matched	Example Peptides
3, 4	AAD25091	*SAG1*	Surface antigen SAG1*	1048	9	K.EIPLESLLPGANDSWWSGVDIK.T; K.SVSSPEVYCTVQVEAER.A
3, 4	AAX38598	*SRS2*	Surface protein SRS2**	760	9	K.LLSEDDGLIVCNESDGEDECEK.N; R.LRPITVNPENNGVTLICGPDGK.A
3, 4	AAG28489	*GRA2*	GRA2 protein	287	4	R.GTVNGQPVGSGYSGYPR.G; R.ESMAAPEDLPGER.Q

3	AAF19184	*MIC3*	Microneme protein Nc-MIC3	219	5	K.NPMCYPTCEEMGGK.D; K.DAECVEDLNAGGSVR.C
3	P90661	*DG1*	GRA7***	116	2	K.LAVPVVGALTSYLVADR.V; R.VLPELTSAEEEGTESIPGK.K

4	AAN16380	*NcMic11*	Microneme protein NcMIC11	137	2	K.STAVEIFK.Q; K.AAIVEGVKPMLPK.L

* also known as Nc-p29 or Nc-p36; ** also known as Nc-p35 or Nc-p43; *** also known as NCDG1 [39].

**Table 2:** Homologues of *T. gondii *proteins identified in more than one fraction

Fraction	Accession	Gene name	Description	MOWSE	Peptides Matched	Example Peptides
3, 4	XP_002369822		SRS domain containing proteins	821	16	K.IDLDPEDLHGHVYLPLVEQVDPMR.L; K.DLGQFGYVPPGDGRDPAGDEVQECK.Y

4, 5	XP_002365950		Glutamine synthetase, putative	168	4	K.IDPPPPADCDAAEVDSPLVR.S; R.TLVDAADLMMVYK.Y + 2 Oxidation (M)

4, 5	EEE20214		20S Proteasome subunit alpha	194	4	K.VEVEVGLIGNDSCGVFK.M; R.IAAVTETIGIAVAGLAADGR.Q

**Table 3:** Homologues of T. *gondii *proteins identified in only one fraction

Fraction	Accession	Gene name	Description	MOWSE	Peptides Matched	Example Peptides
3	EEE22451		Putative uncharacterized protein	837	7	K.LEVGETCTIEMLPQNSK.V; K.HKLEVGETCTIEMLPQNSK.V
3	EEE23072		Putative uncharacterized protein	194	4	R.ATVHPGDTVTMQCPGAISSNPADVSK.Y; R.LILDIEKSEEEVVR.T
3	EEE32684		Surface protein rhoptry protein	135	2	K.SQANQGSPLPPPRPNLLR.R; R.GLMSGVGWVK.R
3	EEE29336		Histone H4	110	2	R.ISGLIYEEIR.G; R.DNIQGITKPAIR.R
3	XP_002370897	*ROP 2*	Rhoptery protein 2	91	3	R.DSGDVILEELFK.R; K.GPSAIVFEATDR.E
3	EEE23774		Ribosomal protein S8	82	2	K.NSIVAIDATPFK.A; K.LDPLLEEQFNTGR.L

4	AAD38419		HSP 60	283	4	K.QVASTTNDIAGDGTTTATLLAR.A; K.TLTHELELVEGLK.F
4	XP_002369317		Proteasome subunit alpha (Type 2)	215	7	R.YNPDIELEDAIHTAILTLK.E; K.EGFEGAMNEHNIEIGVVGEDR.K + Oxidation (M)
4	EEE23454		Proteasome subunit alpha (Type 1)	182	3	K.ELSLDEIQALLDK.M; R.NFESFPGLSPEELELHAMK.A
4	XP_002366589		Proteasome subunit alpha (Type 4)	162	4	K.EDLDVDAALLLAAK.V; K.QEWKEDLDVDAALLLAAK.V
4	EEE19215		Proteasome subunit Beta [(Type 7)	100	2	K.GCAVVLGGVDFK.G; R.VSMAVSVLSQELFK.Y
4	EEE25357		Proteasome subunit alpha (Type 7)	129	2	K.DLVVLAVEK.K; R.LNTATAPSVDYIAK.F

5	EEE30125		Cytosol aminopeptidase putative	142	2	K.LTLFTDDVEAVNR.S R.VVTSFLETLLVELQPDLR.F

Proteins were identified in each fraction by blasting the results of the peptide analysis versus the *N. caninum *and *T. gondii *genomic.

Proteins were identified in each fraction by blasting the results of the peptide analysis versus the *N. caninum *and *T. gondii *genomic databases. **Table 1 **lists protein identified from the *N. caninum *database; **Table 2 ***T. gondii *homologues identified in more than one fraction and **Table 3 ***T. gondii *homologues present only in one of the three fractions. MOWSE scores (for Molecular Weight Search) indicate the likelihood of having correctly identified a specific protein from the molecular weight of the peptides created by its proteolytic digestion and measured with mass spectrometry. The dot (.) in the peptide sequence denotes trypsin cleavage sites.

## Discussion

The primary objective of this work was to identify immunologically (cell mediated) relevant antigens of *N. caninum*, using a combination of proteomics-based and immunological approaches. As CD4^+ve ^T cells are important in disease protection [17] we expanded immune precursors from this population to screen tachyzoite antigens generated through size exclusion fractionation. Selected fractions were then analysed by LC-ESI-MS/MS to catalogue their respective protein profiles. This initial immuno-potency screening of the soluble fractionated antigens was deemed necessary because, whilst proteomic characterisation of an organism provides information on its composition and complexity, it does not always reflect the relative immunological importance of the molecules identified.

We initially demonstrate that size-exclusion fractionation using an aqueous mobile phase is highly reproducible and generates material that is free of detergents and salt concentrations that are incompatible with the *in vitro *CD4^+ve ^test, and for the same reason we opted to use a soluble preparation of the parasite as starting material. A selection based on Western blot reactivity (see additional file 2) would have suggested deeper interrogation of fractions 2 to 7, which correspond to the majority of the proteins present in the original water soluble antigen. However, a comparison of the serological reactivity profiles with the cellular reactivity suggested that only fractions 3, 4 and 5 would benefit from a further analysis. Shotgun proteomics analysis of the selected fractions led to the identification of six *N. caninum *reactive proteins as well as sixteen functional orthologues of *T. gondii *proteins.

Among the six proteins identified from mining the *N. caninum *database and common to both fractions three and four were the surface antigens SAG1 and SRS2 and the dense granule protein GRA2. SAG1 is a tachyzoite glycosylphosphatidylinositol (GPI)-anchored surface molecule [36] thought to be implicated in host cell attachment and invasion [37] and is serologically immunodominant [38]. Recombinant SAG1 immunisation has been attempted with inconsistent results in rodents using a vaccinia virus delivery vector [39], a cDNA prime-protein boost regime [40] or rSAG1 protein only [41]. SRS2 (or Nc-p43) [42] is localised on the surface of *N. caninum *of both bradyzoites and tachyzoites [43], is involved in the host cell invasion process [44] and its neutralisation inhibits parasite attachment and *in vitro *invasion of placental trophoblasts [45]. By homology with *T. gondii *we also identified a second SRS domain-containing protein in fractions 3 and 4, in addition to the *N. caninum *SRS2 protein. SRS-domain containing proteins are considered extremely immunogenic in *Toxoplasma *[46] as well as being present in a large number on the parasite surface, and are thought to facilitate the invasion of multiple host and cell types [47]. Therefore the identification of more than one of these proteins in our *N. caninum *reactive fractions is perhaps not surprising. NcSRS2 has been selected as candidate antigen for vaccination by a number of groups. Rodent challenge with *N. caninum *following vaccination with NcSRS2 demonstrated improved survival [41] reduced transplacental transmission [48] and the development of humoral and cellular immune responses to *N. caninum *tachyzoites [49]. In cattle, NcSRS2 peptide-specific T lymphocytes have been detected *ex vivo *in peripheral blood of infected animals [21]. Baszler et al. [50] also demonstrated the induction of a cell-mediated immune response similar to that induced by the live parasite in animals vaccinated with NcSRS2 in combination with Freund's adjuvant. In addition to surface expressed antigens, we also detected dense granule antigens such as NcGRA2 (p29) and NcGRA7. *Neospora caninum *GRA2 was originally identified by Ellis and collaborators [51] because of its significant amino acid sequence homology (50%) with the GRA2 antigen of *T. gondii*; similarly NcGRA7 (or dense granule protein 1) shows 42% identity with *T. gondii *GRA7 [42]. Dense granule antigens are specialised secretory organelles belonging to the parasitophorous vacuole synthesized at the time of infection and implicated in the cellular invasion process [52] as well as in nutrient acquisition [53]. NcGRA2 is another immunodominant antigen and is recognised by IgM from sera of *N. caninum*-infected cattle [54]. *E. coli *expressed NcGRA2 demonstrated immunogenicity but only partial reduction in foetal infection and pup mortality in a mouse model [55] and Ramamoorthy reported that vaccination of mice with recombinant NcGRA2 induced only partial protection against vertical transmission [56]. Two microneme proteins (Nc-MIC3 and NcMIC11) were also identified in the reactive fractions. Micronemes are secretory organelles which are discharged by exocytosis during the attachment to the host cell surface to facilitate cell invasion [57]. Despite their low molecular weight, microneme proteins could have been eluted in one of the early fractions as protein-complexes, since most of them have putative adhesive functions, are naturally secreted as multiprotein complexes, and immunoprecipitation experiments in *T. gondii *have confirmed that specific microneme proteins form a stable complex within the microneme [58]. In analogy with *T. gondii*, different microneme proteins such as NcMIC11, an ortholog of TgMIC11 [59], NcMIC1 [58] and NcMIC4 [60] have been identified in *N. caninum *but so far only one, Nc-MIC3, has been associated with immunological (serological) reactivity [36]. Use of microneme proteins in vaccination and challenge studies has given contradictory results in rodent models: vaccination with NcMIC4 increased mortality following challenge [61] whereas immunisation with NcMIC1 [62] or NcGRA7 [63] elicited only partial protection. Additional proteins from cellular cytoplasm (rhoptries, ribosomes, HP60), nucleus (hystones) as well as enzymes (proteasome complex, glutamine synthetase, cytosol aminopeptidase) and some additional molecules of unknown function were also found in the reactive fractions by homology with *T. gondii *proteome. The identification of TgROP2 homologue is also promising since immunisation with recombinant NcROP2 in a mouse model has been effective in reducing mortality and cerebral infection [64], in addition to reducing vertical transmission [65] when used in combination with two microneme antigens (NcMIC1 and NcMIC3).

Involvement of proteasome genes in the generation of a protective response to *N. caninum *in mice has been recently suggested by Ellis [66] and HSP60 has been identified as a serologically immunodominant protein [54]. *Neospora caninum *rhoptry antigens have also been identified as serologically immunodominant [67] while in *T. gondii*, some of the rhoptries proteins have been linked to increased virulence [68].

Cell-mediated antigen screening has in the past lead to the identification of parasite fractions capable of being recognised by memory T cells [69,70]. However, because the antigenic components of the parasite were not identified, these previous studies did not allow the selection of specific candidate antigens. Our approach, which combines cellular screening and proteomic characterisation, refines these previous investigations and show that it is possible to streamline the screening of biologically reactive fractions, narrowing the number of molecules of potential interest to a manageable size. Each identified protein can now be investigated to further select those capable of generating the correct *in vivo *immunological response.

## Competing interests

The authors declare that they have no competing interests.

## Authors' contributions

MR, PB and EAI conceived the study and participated in its design and coordination; MR and PB carried out sample collection and cellular assays. ECF performed the serological (ELISA) analysis. NI performed the HPLC separation and the proteomic data collection whereas PB analysed the data. FK participated in the design and the organization of the study. MR, EAI and GE drafted the final version of the manuscript.

All authors read and approved the final manuscript.

## Supplementary Material

Additional file 1 **Liquid Chromatography ElectroSpray Ionisation tandem Mass Spectrometry (LC - ESI-MS/MS) methodology and database mining information**. extended methodological information on the execution of LC - ESI-MS/MS and database mining. Click here for file

Additional file 2 **Western blot reactivity of fractionated NcWSA after separation by size exclusion HPLC probed with a *N. caninum *positive serum**. Western Blot image showing serological reactivity of fractionated *N. caninum *Water-Soluble Antigen, as well as short methodological information. Click here for file

Additional file 3 **SDS PAGE analysis of fractionated *N. caninum *Water-Soluble Antigen after separation by size exclusion**. SDS-PAGE gel image showing proteic composition of HPLC fractionated *N. caninum *Water-Soluble Antigen, as well as short methodological information. Click here for file
