# Supplementary material for: Selection of Neospora caninum antigens stimulating bovine CD4+ve T cell responses through immuno-potency screening and proteomic approaches
Source: Vet Res. 2011 Aug 3;42(1):91. doi: 10.1186/1297-9716-42-91 (PMC3167765; doi:10.1186/1297-9716-42-91)
Supplement: Additional file 1 — Liquid Chromatography ElectroSpray Ionisation tandem Mass Spectrometry (LC - ESI-MS/MS) methodology and database mining information. extended methodological information on the execution of LC - ESI-MS/MS and database mining. [file 1297-9716-42-91-S1.DOC]

**Additional file 1:**

**Title: Liquid Chromatography ElectroSpray Ionisation tandem Mass Spectrometry (LC- ESI-MS/MS) methodology and database mining information.**

**Description:**

**LC-ESI-MS/MS:** Liquid chromatography was performed using an Ultimate 3000 nano-HPLC system (Dionex, Camberley, UK) comprising a WPS-3000 well-plate micro auto sampler, a FLM-3000 flow manager and column compartment, a UVD-3000 UV detector, an LPG-3600 dual-gradient micropump and an SRD-3600 solvent rack controlled by Chromeleon chromatography software (version 6.7, Dionex). A micro-pump flow rate of 246 µL/min^-1^ was used in combination with a cap-flow splitter cartridge, affording a 1/82 flow split and a final flow rate of 3 µL/min^-1^ through a 5 cm × 200 μm ID monolithic reversed phase column (LC Packings, Dionex) maintained at 50 °C. Samples of 4μL were applied to the column by direct injection. Peptides were eluted by the application of a 15 min linear gradient from 8-45% solvent B (80% acetonitrile, 0.1% (v/v) formic acid, all from Sigma, Gillingham, UK) and directed through a 3nL UV detector flow cell. LC was interfaced directly with a 3-D high capacity ion trap mass spectrometer (Esquire HCTplus^TM^, Bruker Daltonics, Coventry, UK) via a low-volume (50 μL/min^-1^ maximum) stainless steel nebuliser (Agilent, cat. no.G1946-20260, Agilent Technologies, Edinburgh, UK) and ESI. Parameters for tandem MS analysis were set as previously described [32].

**Database mining:** Deconvoluted MS/MS data was submitted to an in-house MASCOT server and searched against a) the NCBInr database [33] using alveolata as a taxonomical search parameter and b) a cognate *N. caninum* genomic database [34]. The presentation and interpretation of MS/MS data was performed in accordance with published guidelines [35]. To this end, fixed and variable modifications selected were carbamidomethyl (C) and oxidation (M) respectively and mass tolerance values for MS and MS/MS were set at 1.5Da and 0.5Da respectively. Molecular weight search (MOWSE) scores attained for individual protein identifications were inspected manually and considered significant only if a) two peptides were matched for each protein, and b) each peptide contained an unbroken “*b*” or “*y*” ion series of a minimum of four amino acid residues. A non-redundant list of identified proteins was prepared for each individual fraction and a master list - *N. caninum* reactive protein list (NCRP) generated.
