# Supplementary material for: Selection of Neospora caninum antigens stimulating bovine CD4+ve T cell responses through immuno-potency screening and proteomic approaches
Source: Vet Res. 2011 Aug 3;42(1):91. doi: 10.1186/1297-9716-42-91 (PMC3167765; doi:10.1186/1297-9716-42-91)
Supplement: Additional file 2 — Western blot reactivity of fractionated NcWSA after separation by size exclusion HPLC probed with a N. caninum positive serum. Western Blot image showing serological reactivity of fractionated N. caninum Water-Soluble Antigen, as well as short methodological information. [file 1297-9716-42-91-S2.DOC]

**Additional file 2:**

**Title: Western blot reactivity of fractionated NcWSA after separation by size exclusion HPLC probed with a *N. caninum* positive serum.**

**Description:**


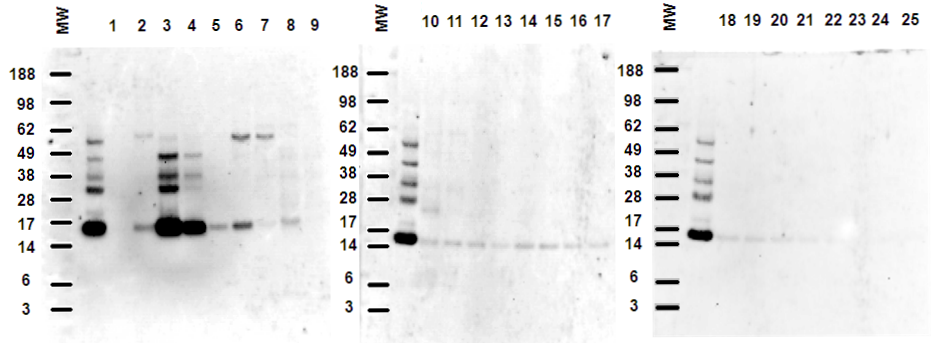


**WSA**

**WSA**

**WSA**

After electrophoretic separation (see supplementary Figure S1) proteins were transfered to a nitrocellulose membrane using a semi-dry blotting system (Trans-Blot® SD Semi-Dry Electrophoretic Transfer Cell, Biorad, Hercules, CA, USA). Prior to immunodetection, the membrane was blocked with 5% non-fat milk in Tris-PBS-Tween 20 (TBST) then was probed with bovine anti-*Neospora* polyclonal antiserum (obtained from the foetus of an experimentally infected dam) and bound antibody was revealed by chemiluminescence using HRP-conjugated rabbit antibovine IgG (Sigma UK) followed by substrate (SuperSignal West Pico Chemiluminescent Substrate, Pierce Thermo Scientific, Rockford, IL, USA) and exposure to radiographic film. Serological reactivity was mainly associated to fractions 2-7. MW: standard of molecular weight; WSA*: N. caninum* Water Soluble antigen; 1-25 denote fraction number.
