# Supplementary material for: Selection of Neospora caninum antigens stimulating bovine CD4+ve T cell responses through immuno-potency screening and proteomic approaches
Source: Vet Res. 2011 Aug 3;42(1):91. doi: 10.1186/1297-9716-42-91 (PMC3167765; doi:10.1186/1297-9716-42-91)
Supplement: Additional file 3 — SDS PAGE analysis of fractionated N. caninum Water-Soluble Antigen after separation by size exclusion. SDS-PAGE gel image showing proteic composition of HPLC fractionated N. caninum Water-Soluble Antigen, as well as short methodological information. [file 1297-9716-42-91-S3.DOC]

**Additional file 3:**

**Title: SDS PAGE analysis of fractionated *N. caninum* Water-Soluble Antigen after separation by size exclusion HPLC.**

**Description:**


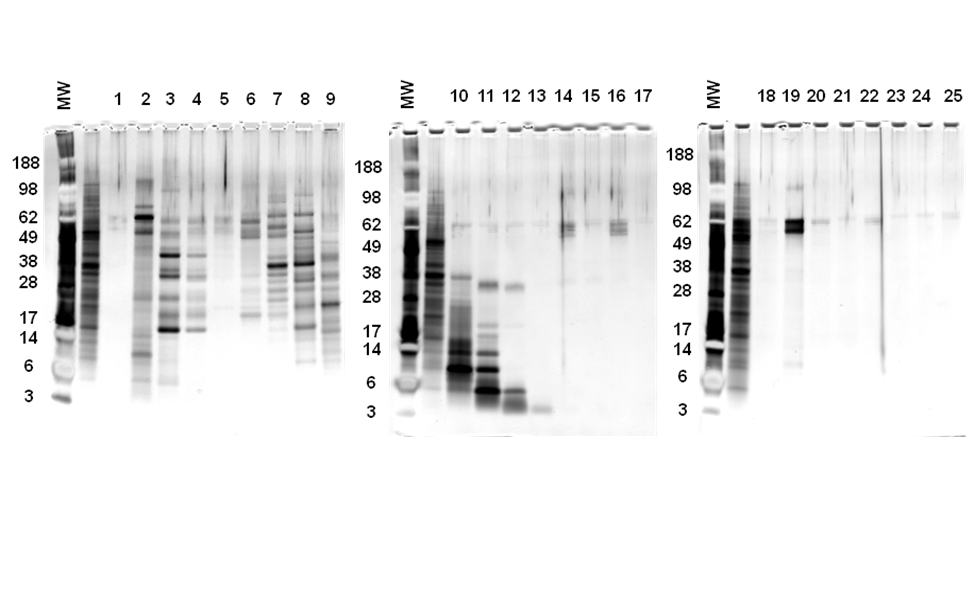


**WSA**

**WSA**

**WSA**

The protein composition of the HPLC separated fractions was analysed by SDS-PAGE. Five μL of each sample or the unfractionated NcWSA were resuspended in 4X NuPAGE® LDS Sample Buffer (Invitrogen, Paisley, UK), heated for 10 min at 70 °C then loaded on a pre cast gradient NuPAGE® Novex Bis-Tris 4-12% mini gels (Invitrogen, UK) and separated under denaturing conditions (NuPAGE® MES buffer, Invitrogen). A pre-stained molecular weight marker (SeeBlue® Plus2, Invitrogen ~~UK~~) (range 188 to 3 KDa) was used to determine molecular mass. Separation was followed by silver staining (Silver Quest™, Invitrogen, ~~UK~~) for band visualization. Images were acquired with an image scanner. *N. caninum* proteins were present with apparent molecular weights ranging from below 188 kDa to approximately 3 kDa. MW: standard of molecular weight; WSA*: N. caninum* Water Soluble antigen; 1-25 denote fraction number.
